# Cox regression and survival analysis from the tauro-urso-deoxycholic trial in amyotrophic lateral sclerosis

**DOI:** 10.3389/fneur.2023.1163855

**Published:** 2023-04-20

**Authors:** Giorgio Reggiardo, Maria Lo Giudice, Stefania Lalli, Gilberto Rinaldi, Alberto Albanese

**Affiliations:** ^1^Department of Biostatistics, Consorzio per Valutazioni Biologiche e Farmacologiche (CVBF), Pavia, Italy; ^2^Department of Neurology, IRCCS Istituto Clinico Humanitas, Milan, Italy; ^3^Bruschettini Srl, Genova, Italy

**Keywords:** amyotrophic lateral sclerosis, tauro-urso-deoxycholic acid, bile acids, disease modification, survival

## Abstract

Recent phase II pilot clinical trials suggested that tauro-urso-deoxycholic acid (TUDCA) might slow functional decline and increase survival in patients with amyotrophic lateral sclerosis (ALS). We performed a multivariate analysis of the original TUDCA cohort to better define the treatment effect and allow comparability with other trials. Linear regression slope analysis showed statistical differences in the decline rate, favoring the active treatment arm (*p*-value < 0.01; −0.262 for the TUDCA group and −0.388 for the placebo group). Mean survival time, estimated by the Kaplan–Meier analysis, showed a 1-month difference, favoring active treatment (log-rank test *p*-value = 0.092). Cox regression analysis demonstrated that placebo treatment was associated with a higher risk of death (*p*-value = 0.055). These data further support the disease-modifying effect of TUDCA monotherapy and raise the question of what could be the additional effect of combining TUDCA with sodium phenylbutyrate.

## Introduction

Amyotrophic lateral sclerosis (ALS) is a rapidly progressive neurodegenerative condition with insufficient therapeutic options. Recent phase II pilot studies reported that the administration of tauro-urso-deoxycholic acid (TUDCA) could slow functional decline and increase survival in patients with ALS (1, 2). Among approved disease-modifying therapies for ALS, only riluzole has been shown to improve tracheostomy-free survival (3). These observations reinforced interest in the potential efficacy of TUDCA as a disease modifier for ALS. Considering that the analysis of the original TUDCA cohort used a univariate approach on individual response variables, there is a need to perform a multivariate approach to increase sensitivity, improve detection of the treatment effect, and allow comparability with other trials.

We reviewed the original phase II TUDCA dataset and present here a final intention-to-treat analysis of survival data.

## Materials and methods

The TUDCA phase II study (NCT00877604) was conducted at three Italian centers. Protocol approval was provided by a central institutional review board for all trial sites. Participants provided written informed consent before entering the trial. Detailed methods have been published (1). Briefly, adults with definite ALS (revised El Escorial criteria) who were ≤18 months from symptom onset were randomized 1:1 to receive daily TUDCA (1 g twice daily) or placebo by mouth. The total study duration was 66 weeks, including a 12-week lead-in period and a 54-week treatment period.

A *post-hoc* analysis of the entire trial dataset was performed. This analysis included the assessment of change from the baseline and multivariate Cox regression. The slopes of the two linear regression equations of ALSFRS-R scores during the treatment period (ALSFRS-R mean scores over time) were compared using a generalized linear mixed effects model with fixed effects for the time, treatment group, and their interaction (4).

Mean and median survival durations and 95% confidence intervals (CI) were estimated by using the Kaplan–Meier approach. The median survival time was reported only if the estimated survival probability reached 50%. The hazard ratio (HR) of death was estimated using a Cox proportional hazards model with age at randomization and baseline ALSFRS-R total score as covariates. Analyses of overall results began at randomization, with censoring defined as the earliest occurrence between death and the last available follow-up. Survival data until 66 weeks were also collected. Hazard ratios for TUDCA treatment vs. placebo were obtained with 95% confidence intervals, and the *p*-value was used in testing the significance of treatment differences. All analyses were done with SAS (version 9.2; Cary, NC, USA). All *p*-values were two-sided and were regarded as significant if <0.05.

## Results

ANCOVA analysis showed significant between-group differences in ALSFRS-R total score change from the baseline, favoring subjects randomized to TUDCA (*p* = 0.016). The estimated marginal mean decrement score, corrected for the ALSFRS-R score at the baseline, was 36.8% in the TUDCA group vs. a decrement of 53.0% in the placebo group. This indicated a 16.2% difference, favoring TUDCA over placebo.

Regression line slopes of the two treatment arms showed a significant difference in decline rate, favoring TUDCA (*p*-value < 0.01; −0.262 for TUDCA and −0.388 for placebo).

In the overall survival Kaplan–Meier analysis, encompassing all randomized participants (N = 34), mean (95% CI) survival time was 15.3 months (15.2–15.5) in the TUDCA arm and 14.3 months (12.9–15.6) in the placebo arm. The log-rank test indicated no significant difference between the two treatment arms (*p*-value = 0.092).

**Figure 1:**
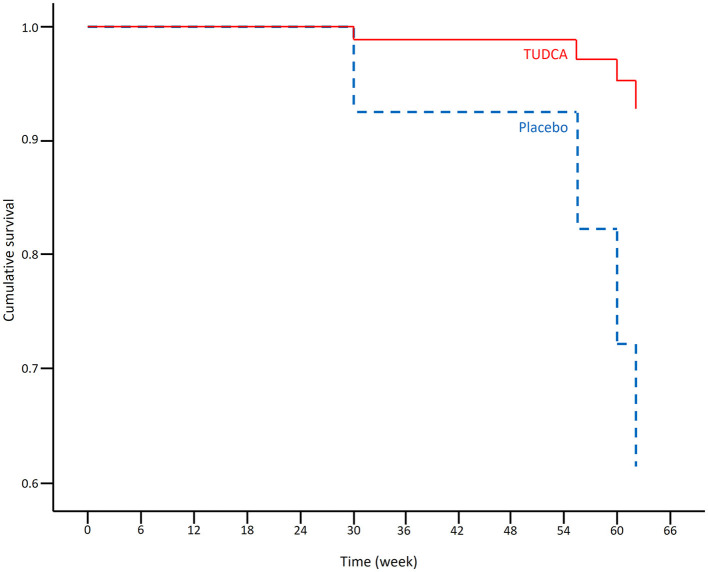
Cox regression survival plot estimating the probability of survival in the placebo (blue dashed line) and TUDCA (red solid line) arms (HR = 0.051, 95% CI = 0.002–1.059).

The results of the Cox regression model for time to death in all patients are shown in Table 1. There were non-significant effects of the ALSFRS-R total score at the baseline and the treatment group on survival: Placebo (*p*-value = 0.055) and lower ALSFRS-R total score at the baseline (*p*-value = 0.128) were associated with a higher risk of death. The Cox regression survival plot describing the estimated model is shown in Figure 1.

**Table 1:** Cox proportional hazards analysis of time to death in the phase II TUDCA trial according to three different subgroup analyses: treatment, baseline ALSFRS-R, and age.

**Cox regression model**	**B**	**SE**	**Wald test**	**df**	***p*-value**	**HR**	**95.0% CI for HR**	
							**Lower**	**Upper**
TUDCA vs. placebo	−2.979	1.550	3.697	1	0.055	0.051	0.002	1.059
Baseline ALSFRS-R	−0.317	0.208	2.322	1	0.128	0.729	0.485	1.095
Age (years)	0.047	0.048	0.932	1	0.334	1.048	0.953	1.152
Un-adjusted model								
TUDCA vs. placebo	−1.903	1.167	2.658	1	0.103	0.149	0.015	1.469

Treatment group allocation influenced survival to an almost significant level, whereas functional status and age did not influence survival. B, regression coefficients; CI, confidence interval; df, degree of freedom; HR, hazard ratio; SE, standard error.

## Discussion

*Post-hoc* analysis of data from the phase II TUDCA trial shows that, after correction for age and basal ALSFRS-R score, participants in the active arm had a 95% lower risk of mortality compared to participants in the placebo arm (Table 1). This is in keeping with a less steep slope in the active treatment. It is important to note that *post-hoc* subgroup analysis has limitations as the subgroups are small, and potential confounding differences among the groups were only controlled to a limited extent, as covariates of age at randomization, pre-baseline ALSFRS-R slope, and baseline ALSFRS-R total score were comparable.

Cox regression is a widely adopted model for the analysis of survival data, providing hazard ratios for variables included in the model. This can support decision-making by clinicians. Similarly, Cox proportional hazards regression can provide an effect estimate by quantifying the difference in survival between patient groups and can adjust for confounding effects of other variables. These two statistical measures are often combined for the study of progressive neurodegenerative diseases (5).

An added value of this *post-hoc* analysis is to provide a longer follow-up to the originally published data (1) and additionally to compare the results of this phase II trial with a recent *post-hoc* analysis of the phase II CENTAUR trial (NCT03127514), which tested a combination of TUDCA (1 g twice daily) and NaPB (3 g twice daily) in ALS treatment (6). The CENTAUR trial showed a 0.56 HR, favoring the active treatment arm (6). The between-group ALSFRS-R total score change from the baseline difference in the CENTAUR trial indicated an 8.57% difference (18.60% decrease in the active treatment arm vs. 27.17% decrease with placebo) (2). The results of the two-phase II TUDCA trials showed remarkable similarities: There is a 7.63% better performance in the TUDCA trial compared to the CENTAUR trial, which is, however, insufficient to draw conclusions of clinical significance. The question of whether the addition of NaPB to TUDCA is advantageous, neutral, or disadvantageous can only be addressed by comparing the results of ongoing phase III studies (NCT03800524 and NCT05021536), one based on TUDCA alone, and the other on TUDCA in combination with NaPB. This coincidental parallelism may provide stronger answers if both datasets will be made publicly available for comparison.

## Data availability statement

The data analyzed in this study is subject to the following licenses/restrictions: data is not available because subjects enrolled in the study did not provide the consent to share data publicly. Requests to access these datasets should be directed to AA, alberto.albanese@unicatt.it.

## Ethics statement

The studies involving human participants were reviewed and approved by Carlo Besta Neurological Institute Ethics Committee. The patients/participants provided their written informed consent to participate in this study.

## Author contributions

GRe: data analysis and statistics and manuscript review and critique. ML: first draft and manuscript review and critique. SL: data acquisition. GRi: manuscript review and strategic analysis. AA: conceptual idea and manuscript review and critique. All authors contributed to the article and approved the submitted version.
